# Synthesis, crystal structure and computational analysis of 2,7-bis­(4-chloro­phen­yl)-3,3-dimethyl-1,4-diazepan-5-one

**DOI:** 10.1107/S2056989023010162

**Published:** 2023-11-30

**Authors:** Shanmugasundaram Akila, Thankakan Vidhyasagar, John Peter Winfred Jebaraj, Aravazhi Amalan Thiruvalluvar, Krishnan Rajeswari

**Affiliations:** a Department of Chemistry, Annamalai University, Annamalai Nagar 608 002, Tamil Nadu, India; b Department of Chemistry, St. John’s College, Palayamkottai 627 002, Tamil Nadu, India; c Principal (Retired), Kunthavai Naacchiyaar Government Arts College for Women (Autonomous), Thanjavur 613 007, Tamil Nadu, India; d PG & Research Department of Chemistry, Government Arts College, Chidambaram 608 102, Tamil Nadu, India; Universidad de Los Andes, Venezuela

**Keywords:** synthesis, X-ray crystal structure, C—H⋯O and N—H⋯O hydrogen bonds, C—Cl⋯π (ring) inter­actions, 1,4-diazepane derivative, chair conformation, DFT, Hirshfeld surface analysis, 3ERT protein, mol­ecular docking

## Abstract

In the title compound, C_19_H_20_Cl_2_N_2_O, the seven-membered 1,4-diazepane ring adopts a chair conformation while the 4-chloro­phenyl substituents adopt equatorial orientations. The chloro­phenyl ring at position 7 is disordered over two positions [site occupancies 0.480 (16):0.520 (16)]. The dihedral angle between the two benzene rings is 63.0 (4)°. The methyl groups at position 3 have an axial and an equatorial orientation. The compound exists as a dimer exhibiting inter­molecular N—H⋯O hydrogen bonding with *R* ^2^_2_(8) graph-set motifs. The crystal structure is further stabilized by C—H⋯O hydrogen bonds together with two C—Cl⋯π (ring) inter­actions. The geometry was optimized by DFT using the B3LYP/6–31 G(d,p) level basis set. In addition, the HOMO and LUMO energies, chemical reactivity parameters and mol­ecular electrostatic potential were calculated at the same level of theory. Hirshfeld surface analysis indicated that the most important contributions to the crystal packing are from H⋯H (45.6%), Cl⋯H/H⋯Cl (23.8%), H⋯C/C⋯H (12.6%), H⋯O/O⋯H (8.7%) and C⋯Cl/Cl⋯C (7.1%) inter­actions. Analysis of the inter­action energies showed that the dispersion energy is greater than the electrostatic energy. A crystal void volume of 237.16 Å^3^ is observed. A mol­ecular docking study with the human oestrogen receptor 3ERT protein revealed good docking with a score of −8.9 kcal mol^−1^.

## Abstract 2

The seven-membered 1,4-diazepane ring adopts a chair conformation with the 4-chloro­phenyl groups in equatorial orientations. The title compound possesses a docking score of −8.9 kcal mol^−1^ with the human oestrogen receptor 3ERT protein.

## 1. Chemical context

Quite a few reports have long established that 1,4-diazepane derivatives (Sethuvasan *et al.*, 2016 ▸; Maheshwaran *et al.*, 2015 ▸) are chemically (Baliah *et al.*, 1978 ▸; Thennarasu & Perumal, 2002 ▸) and biologically (Murthy & Knaus, 1999 ▸; Wolkinger *et al.*, 2009 ▸) significant motifs. In the view of widespread applications of 1,4-diazepane in the synthetic and medicinal fields, we report here the synthesis, crystal structure and computational analysis of 2,7-bis­(4-chloro­phen­yl)-3,3-dimethyl-1,4-diazepan-5-one (I).

## 2. Structural commentary

**Figure 1:**
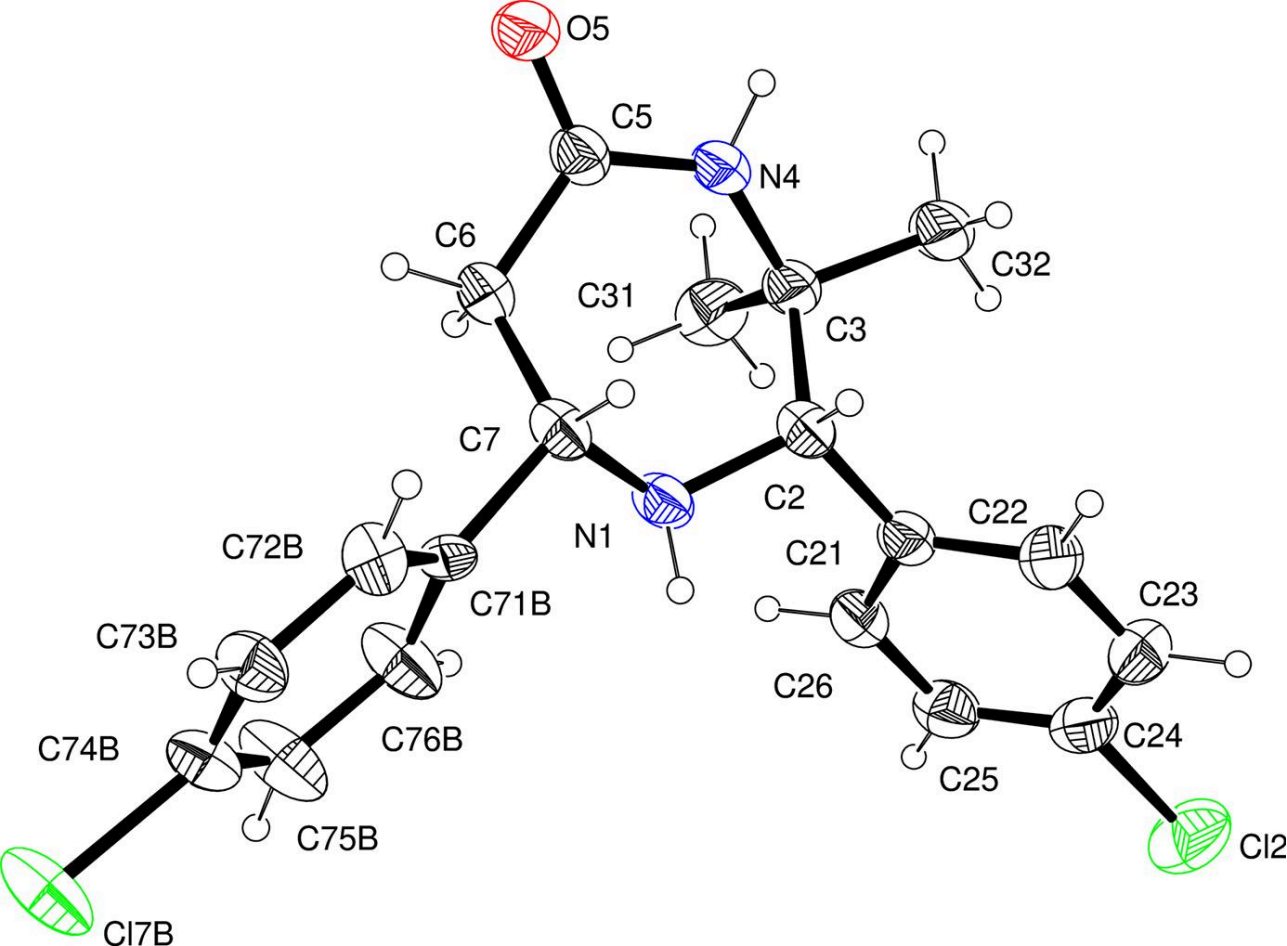
View of the mol­ecular structure of (I), showing 30% probability displacement ellipsoids (arbitrary spheres for the H atoms). The minor component of the disorder is not shown for clarity.

In the title compound, which crystallizes in the monoclinic crystal system, space group *P*2_1_/*n*, with *Z* = 4 (Fig. 1 ▸), the seven-membered 1,4-diazepane (N1/C2/C3/N4/C5/C6/C7) ring is in a chair conformation and exhibits puckering parameters (Cremer & Pople, 1975 ▸) *Q*_T_ = 0.721 (2) Å, *q*2 = 0.259 (2) Å, *q*3 = 0.673 (2) Å, φ(2) = −157.2 (4)° and φ(3) = 5.16 (14)°. The spherical polar angle θ(2) = 21.09 (13)°. The displacements of atoms N1, C2, C3, N4, C5, C6, and C7 from the least-squares plane defined by C2/C3/C6/C7 are −0.7164 (20), −0.0283 (9), 0.0226 (7), 0.9539 (26), 0.8620 (28), −0.0232 (8) and 0.0288 (9) Å, respectively, confirming the chair conformation of the 1,4-diazepane ring. The dihedral angles between the best plane of the diazepane ring (C2/C3/C6/C7) and the planar 4-chloro­phenyl rings [C21–C26 and C71*B*–C76*B*] are 88.1 (1)° and 82.7 (3)°, respectively. The sum of the bond angles at the nitro­gen atom N1 is 332.2°, indicating a pyramidal geometry at N1. The sum of the bond angles at N4 is 356.2°, indicating a planar configuration at N4. As evident from torsion angles N4—C3—C2—C21 [−166.89 (13)°] and C5—C6—C7—C71*B*[163.1 (4)°], the 4-chloro­phenyl rings at C2 and C7 both occupy the equatorial positions of the 1,4-diazepane chair ring. One of the methyl groups at C3, occupies the axial position [N1—C2—C3—C31 = −52.76 (19)°] while the other methyl [N1—C2—C3—C32 = −174.79 (15)°] occupies the equatorial position. The 4-chloro­phenyl ring at C7 is disordered over two positions [C71*A*–C76*A* (minor) and C71*B*–C76*B* (major) components with an inter­planar angle of 12.2 (4)°; refined occupancy ratio of 0.480 (16):0.520 (16)]. The main residue disorder is 29%. 
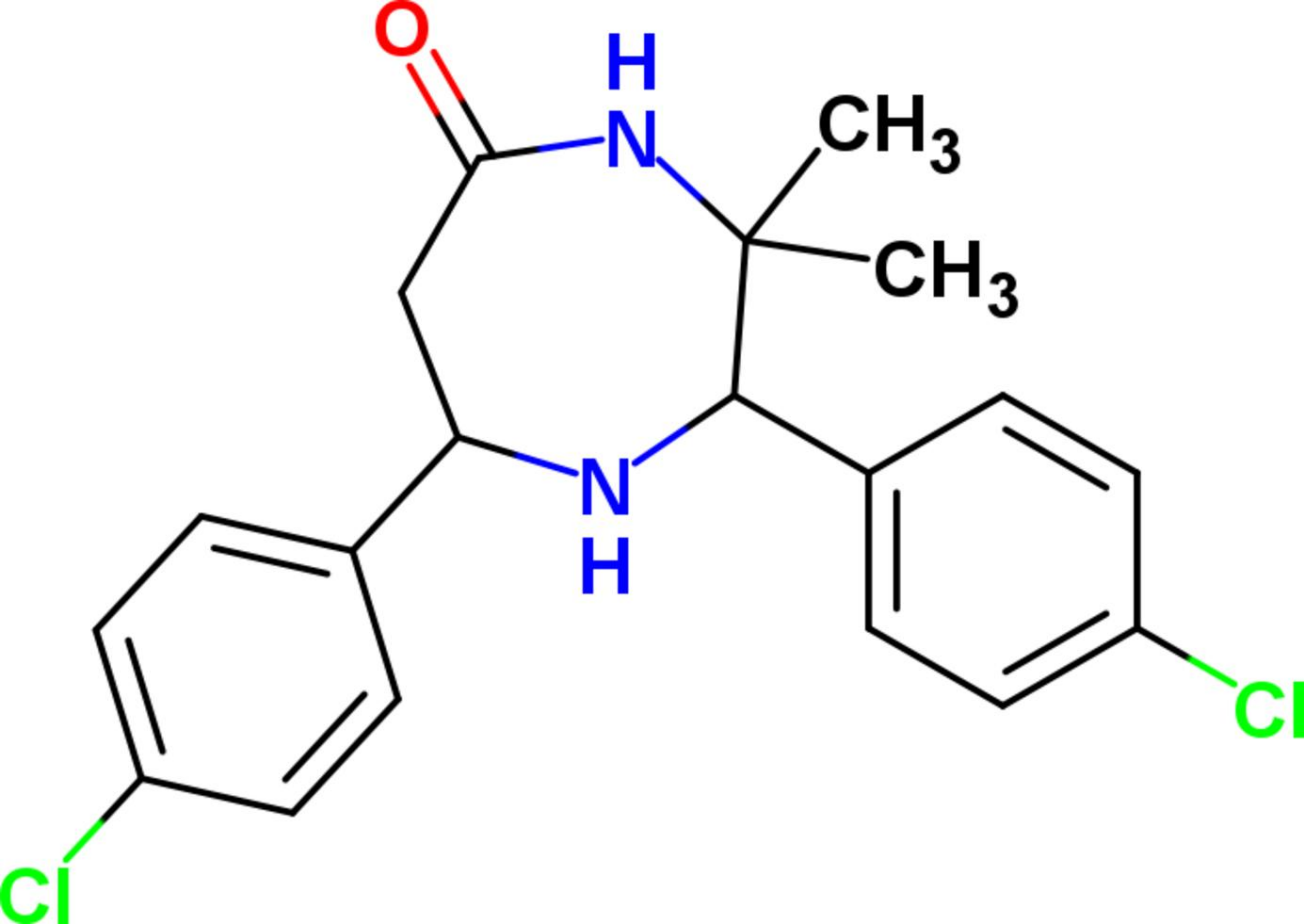


## 3. Supra­molecular features

**Figure 2:**
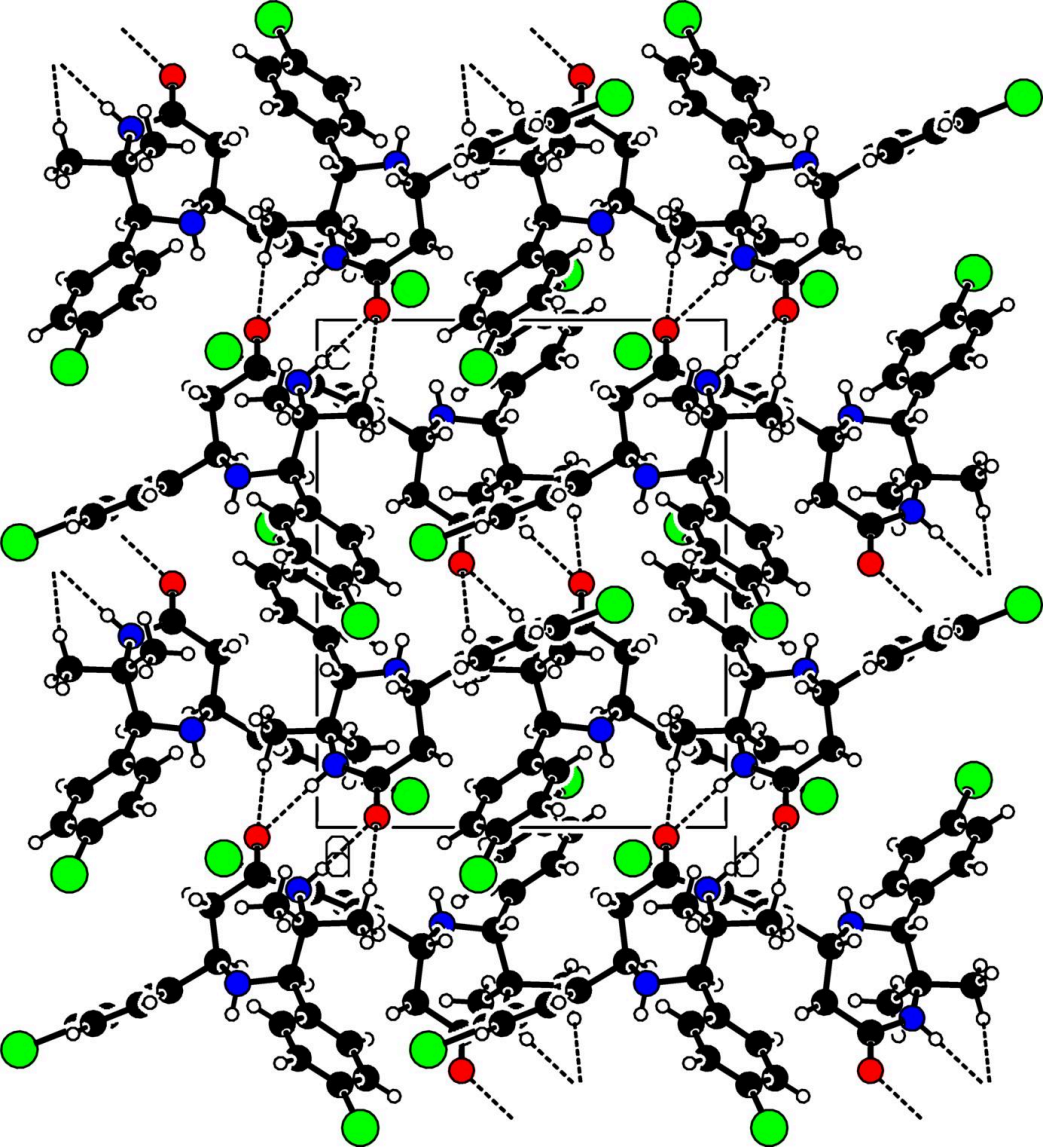
A partial packing diagram of the title compound viewed along the *a* axis showing the C—H⋯O and N—H⋯O hydrogen-bond inter­actions (dashed lines).

In the crystal, N4—H4⋯O5^i^ hydrogen-bonding inter­actions (Fig. 2 ▸, Table 1 ▸) form dimers with an 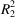 (8) graph-set motif. The mol­ecules are further linked by C32—H32*A*⋯O5^i^ and C73*B*—H73*B*⋯O5^ii^ hydrogen bonds and C—Cl⋯π inter­actions [C24—Cl2⋯*Cg*3(− 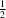 + *x*, 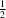 − *y*, 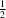 + *z*): C24—Cl2 = 1.744 (2) Å, Cl2⋯*Cg*3 = 3.641 (5) Å, C24⋯*Cg*3 = 4.946 (8) Å and C24—Cl2⋯*Cg*3 = 129.99 (11)°; C74*A*—Cl7*A*⋯*Cg*1(1 − *x*, 1 − *y*, 1 − *z*): C74*A*—Cl7*A* = 1.756 (12) Å, Cl7*A*⋯*Cg*1 = 3.772 (8) Å, C74*A*⋯*Cg*1 = 5.467 (12) Å and C74*A*—Cl7*A*⋯*Cg*1 = 161.7 (6)°; *Cg*1 and *Cg*3 are the centroids of the C21–C26 and C71*A*–C76*A* rings, respectively]

## 4. DFT Studies

The theoretical optimized structure of (I) for the disordered mol­ecule with higher site occupancy in the gas phase was computed using *Gaussian 09W*, Revision A.02 (Frisch *et al.*, 2009 ▸) by applying the B3LYP/6-31G(d,p) level basis set. The optimized structure, HOMO and LUMO energies, and mol­ecular electrostatic potential were generated using *GaussView 5.0* (Dennington *et al.*, 2009 ▸). Comparison of calculated geometrical parameters with those of the experimental results revealed that they are generally in good agreement (Table 2 ▸). The slight variations between the geometrical parameters observed for the gas phase (theoretical) and those of the solid phase (experimental) are quite explicable.

**Figure 3:**
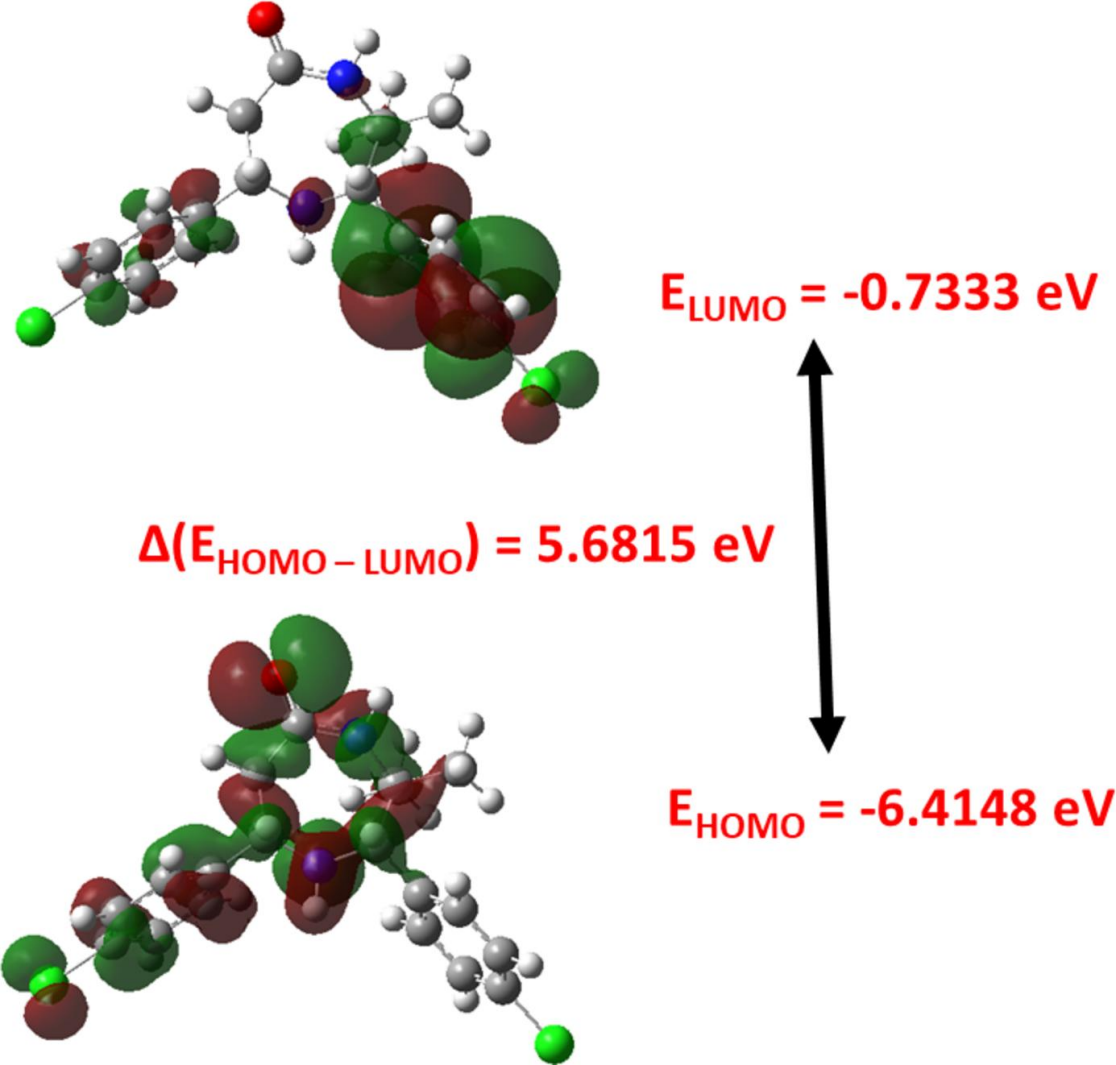
HOMO and LUMO of (I).

The electron density in highest occupied mol­ecular orbital and lowest unoccupied mol­ecular orbital computed are shown in Fig. 3 ▸. In the HOMO, the electron density largely resides over the diazepanone ring and the phenyl ring at C7 whereas in the LUMO, the electron density is delocalized and largely resides over the phenyl ring at C2. The energies of frontier mol­ecular orbitals *E*_HOMO_ and *E*_LUMO_ are −6.4148 eV and −0.7333 eV, respectively. The energy gap Δ*E* (E_LUMO_ - E_HOMO_) is 5.6815 eV. The electron affinity (*A* = -E_LUMO_ = 0.7333 eV) and ionization potential (*IP* = -*E*_HOMO_ = 6.4148 eV) were used to calculate the electronegativity (χ = 3.5740 eV), chemical hardness (η = 2.8407 eV) and chemical softness (*S* = 0.1760 eV). From the values of chemical hardness and the high energy gap, it is understood that the mol­ecule is chemically hard and less polarizable.

**Figure 4:**
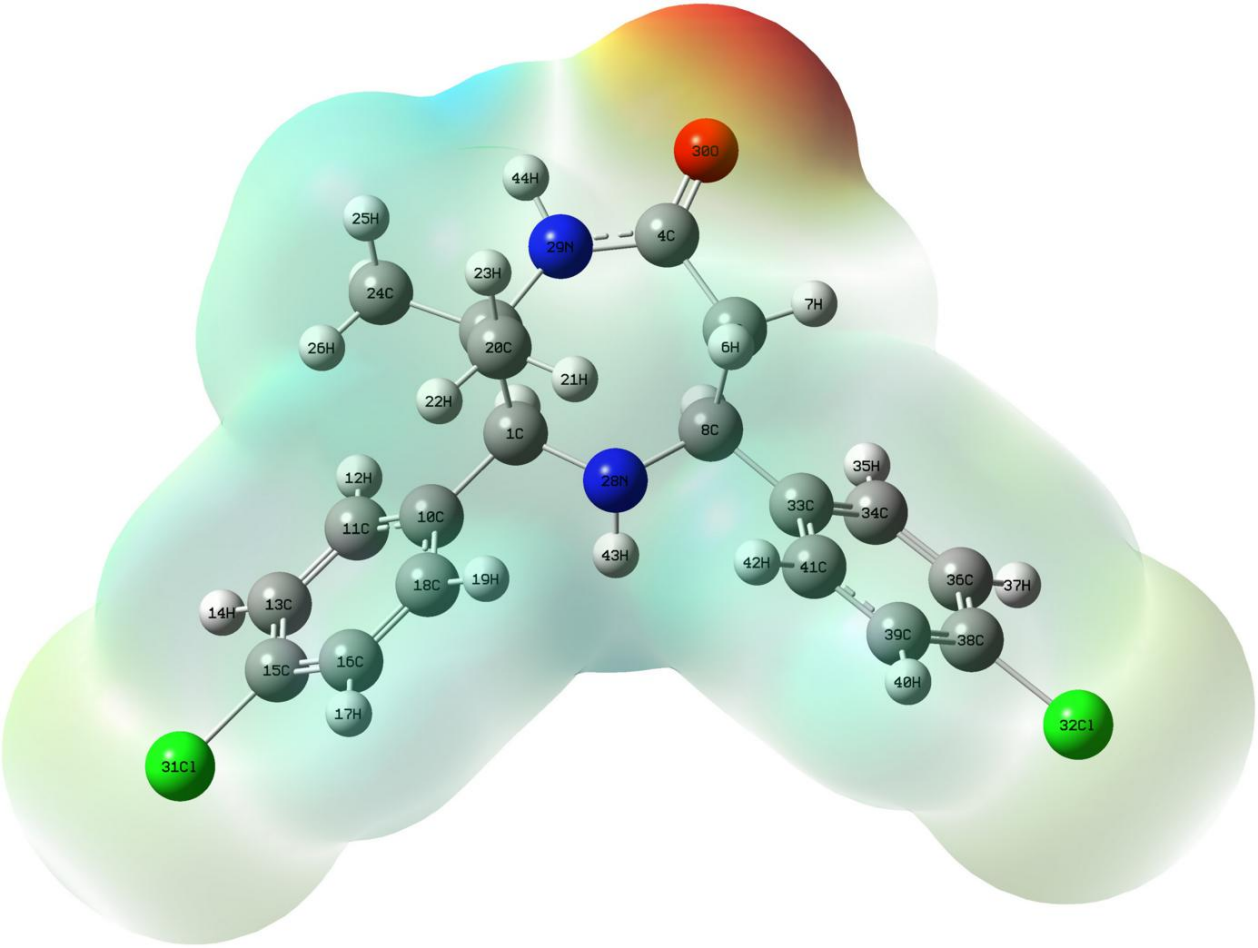
Mol­ecular electrostatic potential surface diagram of (I).

The mol­ecular electrostatic potential (MEP) surface (Fig. 4 ▸) provides information about the reactive sites of (I). The red region on the MEP surface over the carbonyl oxygen atom indicates an electron-rich centre with partial negative charge, which is vulnerable to electrophilic attack, whereas the yellow region over both the chlorine atoms shows a less electron-rich region and the pale-blue region spread all over the mol­ecule indicates the less electron-deficient region (Politzer & Murray, 2002 ▸).

## 5. Hirshfeld surface and two-dimensional fingerprint plots

**Figure 5:**
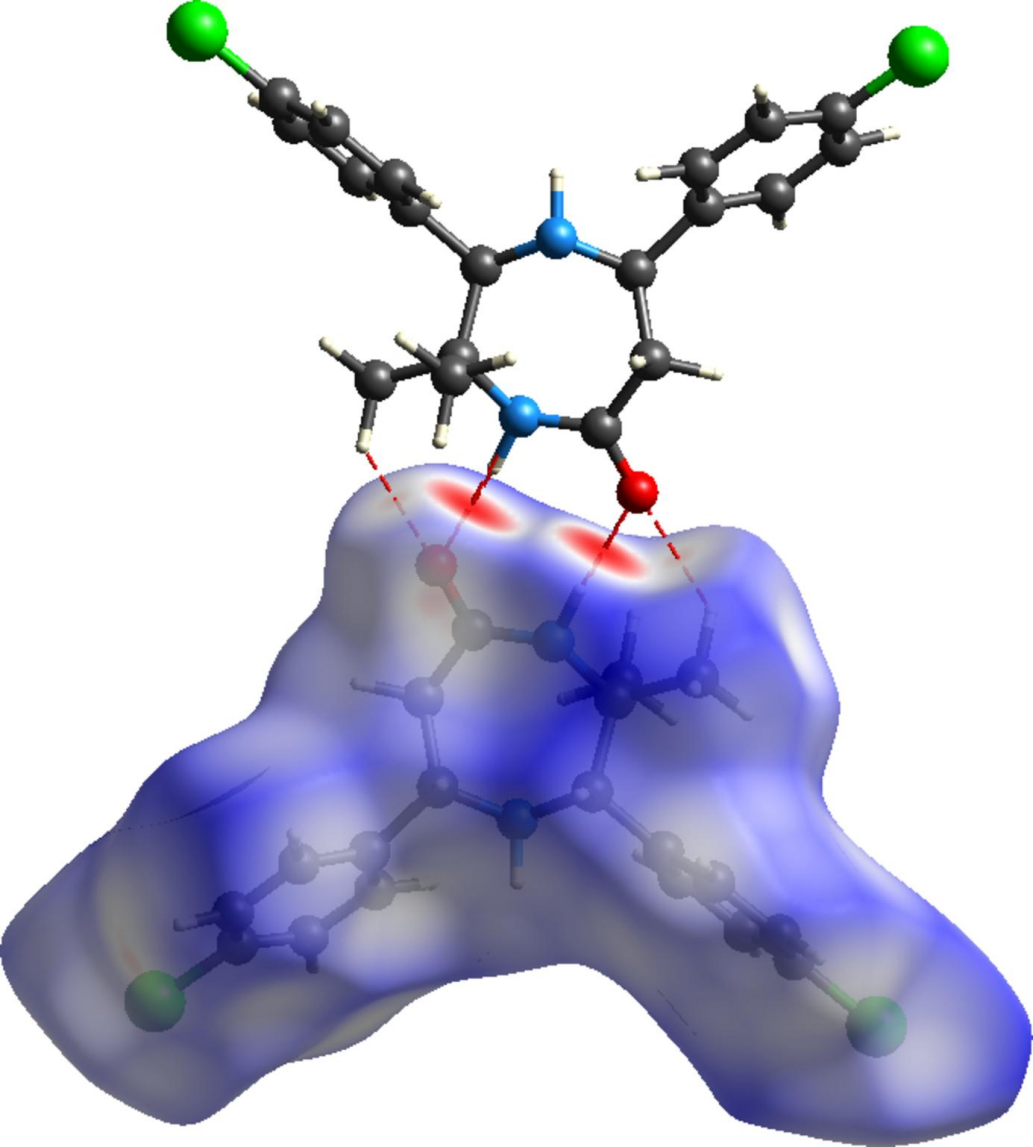
Hirshfeld surface for (I) showing hydrogen-bonding inter­actions with a neighbouring mol­ecule. The minor component of the disorder is not shown for clarity.

The Hirshfeld surface and two-dimensional fingerprint plots including all orientations of the disordered mol­ecule were generated using *CrystalExplorer 21.5* (Spackman *et al.*, 2021 ▸) to study the mol­ecular inter­actions with enhanced details (see also Fig. S1 in the supporting information). The Hirshfeld surface plotted over *d*_norm_ in the range −0.5371 to 1.5160 a.u. is shown in Fig. 5 ▸. The intense red spots indicating contacts shorter than the sum of van der Waals radii seen between N—H⋯O represent the shortest inter­molecular contacts between nearest mol­ecules while the other red spots indicated the inter­actions between C—H⋯O. The blue region denotes the longest inter­actions and the white medium-length inter­actions.

**Figure 6:**
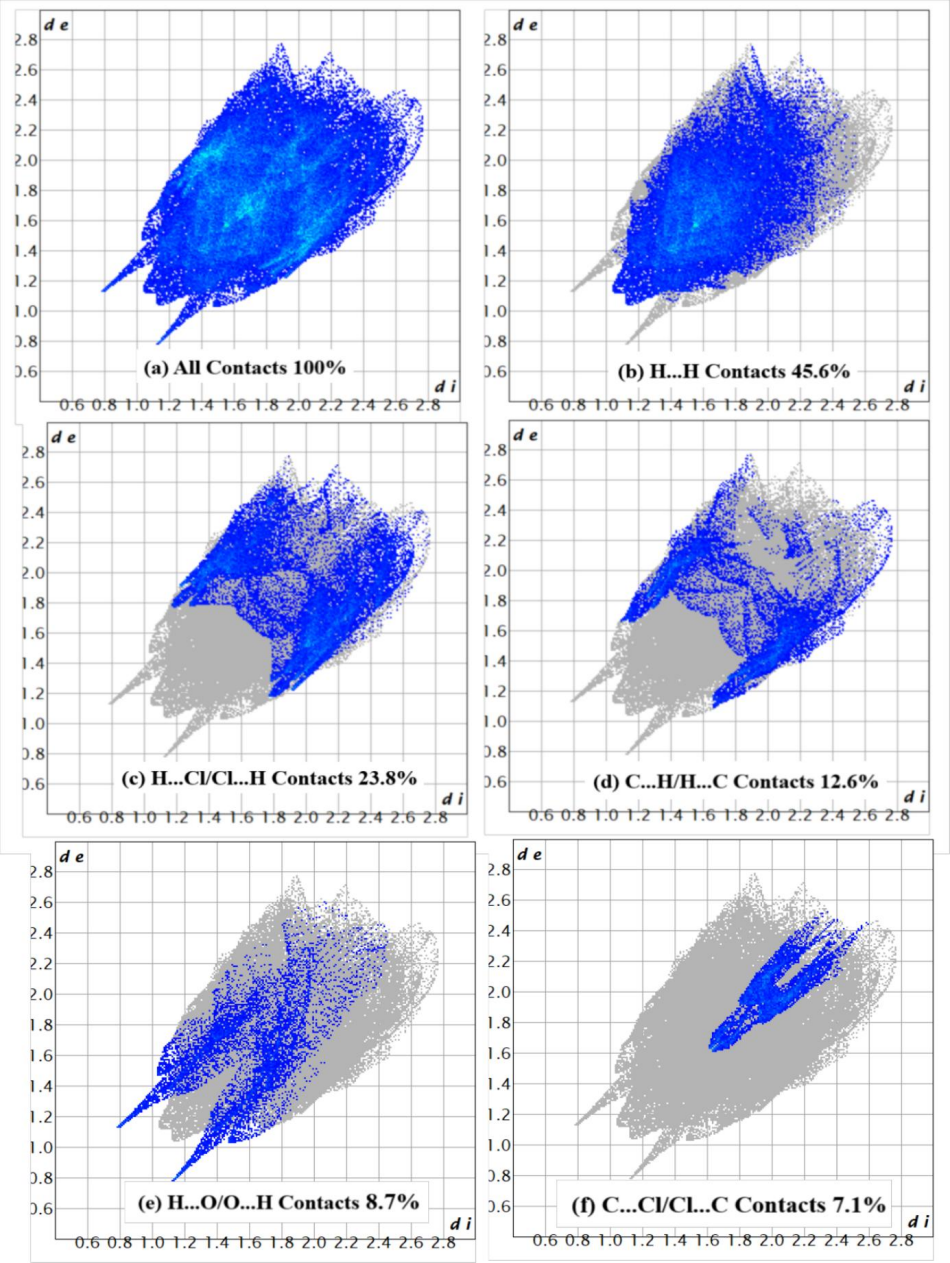
Two-dimensional-fingerprint plots for (I).

The two-dimensional-fingerprint plots (Fig. 6 ▸) indicate that the most important contributions to the crystal packing are from H⋯H (45.6%), Cl⋯H/H⋯Cl (23.8%), H⋯C/C⋯H (12.6%), H⋯O/O⋯H (8.7%) and C⋯Cl/Cl⋯C (7.1%) inter­actions.

## 6. Crystal void analysis

**Figure 7:**
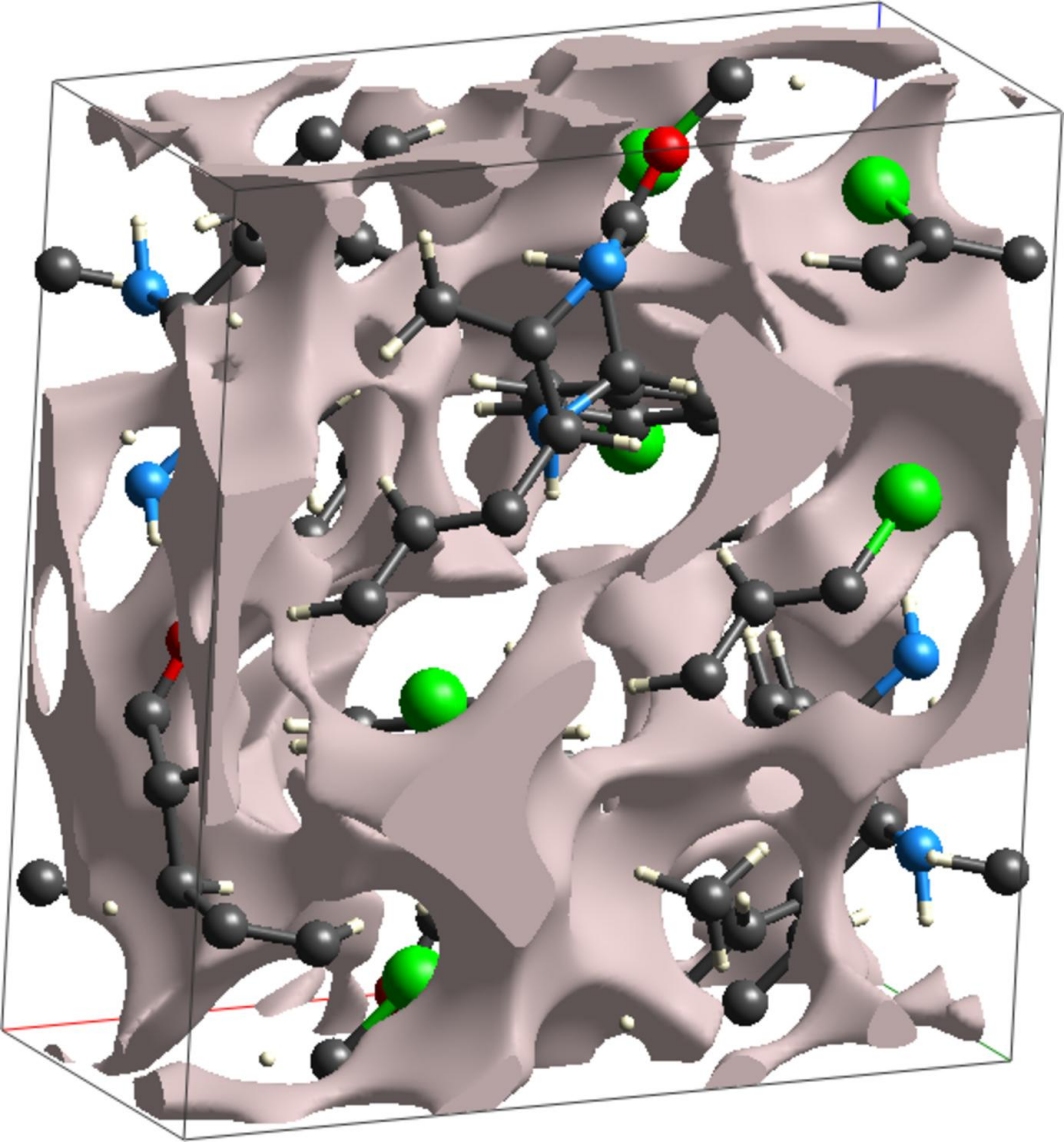
Crystal voids in (I).

The effectiveness of the packing of mol­ecules in the unit cell of the crystal can be assessed with void analysis. The crystal void surfaces, *i.e.* the empty region of the crystal structure, define the isosurface of the procrystal electron density, and are generally calculated for the whole unit cell (Turner *et al.*, 2011 ▸). The spatial void volume of the crystal of (I) (Fig. 7 ▸, see also Fig. S2 in the supporting information) in the unit cell was calculated (including all the orientations of the disordered mol­ecule with partial site occupancies) to be 237.16 Å^3^, *i.e.*, 12.46%, of the crystal volume, which shows the mechanical strength of the crystal is high.

## 7. Inter­action energies and Energy frame works

**Figure 8:**
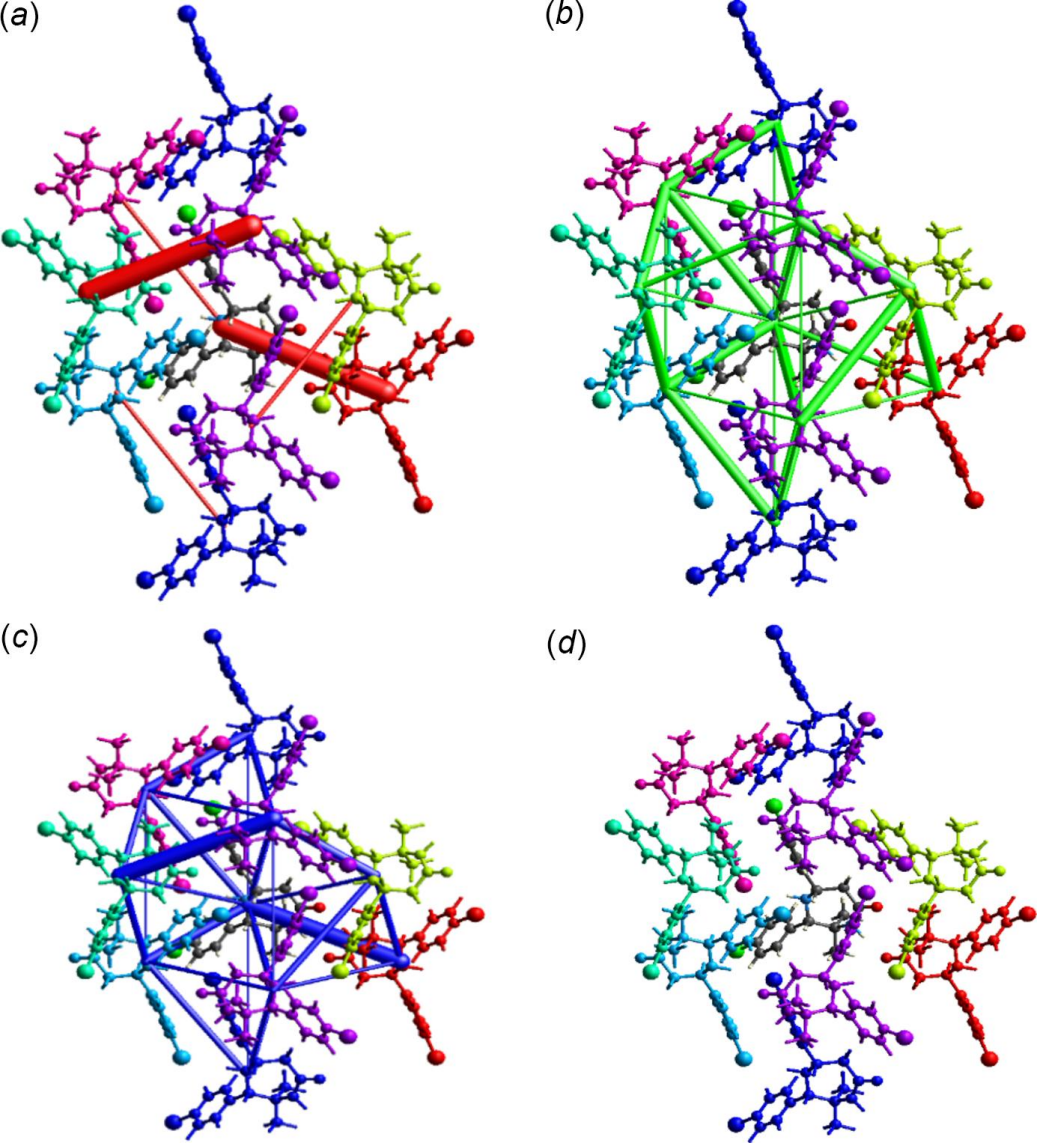
Graphical representation of energy frameworks of (I): (*a*) electrostatic energy, (*b*) dispersion energy, (*c*) total energy and (*d*) colour-coded diagram of (I).

The inter­molecular inter­action energies were calculated for the disordered model with the higher site occupancy using CE-HF/6-31G(d,p) energy model in *CrystalExplorer* (Mackenzie *et al.*, 2017 ▸; Turner *et al.*, 2015 ▸). A cluster of mol­ecules is generated with respect to a selected central mol­ecule within a radius of 3.8 Å and the inter­action energies computed (see also Fig. S3 in the supporting information). The calculated inter­action energies are shown in the form of the graphical-cylindrical representation known as energy frameworks (Fig. 8 ▸). The frameworks constructed for *E*_ele_ (red cylinders), *E*_dis_ (green cylinders) and *E*_total_ (blue cylinders) help to visualize the supra­molecular architecture of (I). From the energy framework representation, it is evident that the dispersion energy of the title compound is greater than the electrostatic energy.

## 8. Mol­ecular docking study

**Figure 9:**
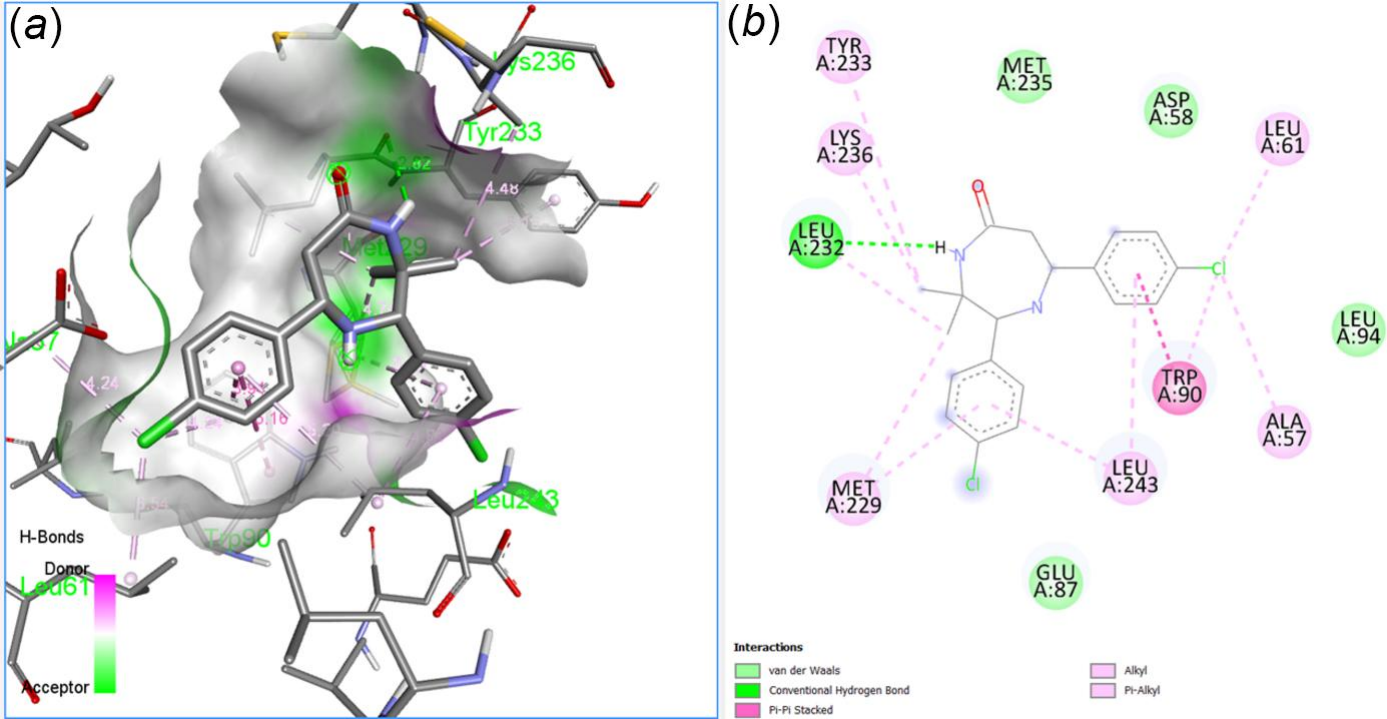
Mol­ecular docking: (*a*) three-dimensional and (*b*) two-dimensional views of the inter­action of (I) with 3ERT protein.

A mol­ecular docking study was performed to examine the binding affinity of the title ligand with the human oestrogen receptor alpha (hER alpha) protein, for which the structural coordinates were retrieved from the Protein Data Bank (https://www.rcsb.org; PDB ID: 3ERT) in CIF format. The input file for the ligand was obtained by converting the CIF file (containing only the major component of the disorder) to pdb format using *Mercury* (version 2023.2.0; Macrae *et al.*, 2020 ▸) and the docking studies carried out using the *PyRx* virtual screening tool (version 1.0; Dallakyan & Olson, 2015 ▸) and the results viewed using Discovery Studio Visualizer (v21.1.0.20298; Biovia, 2017 ▸) software. The mol­ecular docking of (I) with 3ERT protein is shown in Fig. 9 ▸, revealing a good binding affinity, with a score of −8.9 kcal mol^−1^.

## 9. Database survey

**Figure 10:**
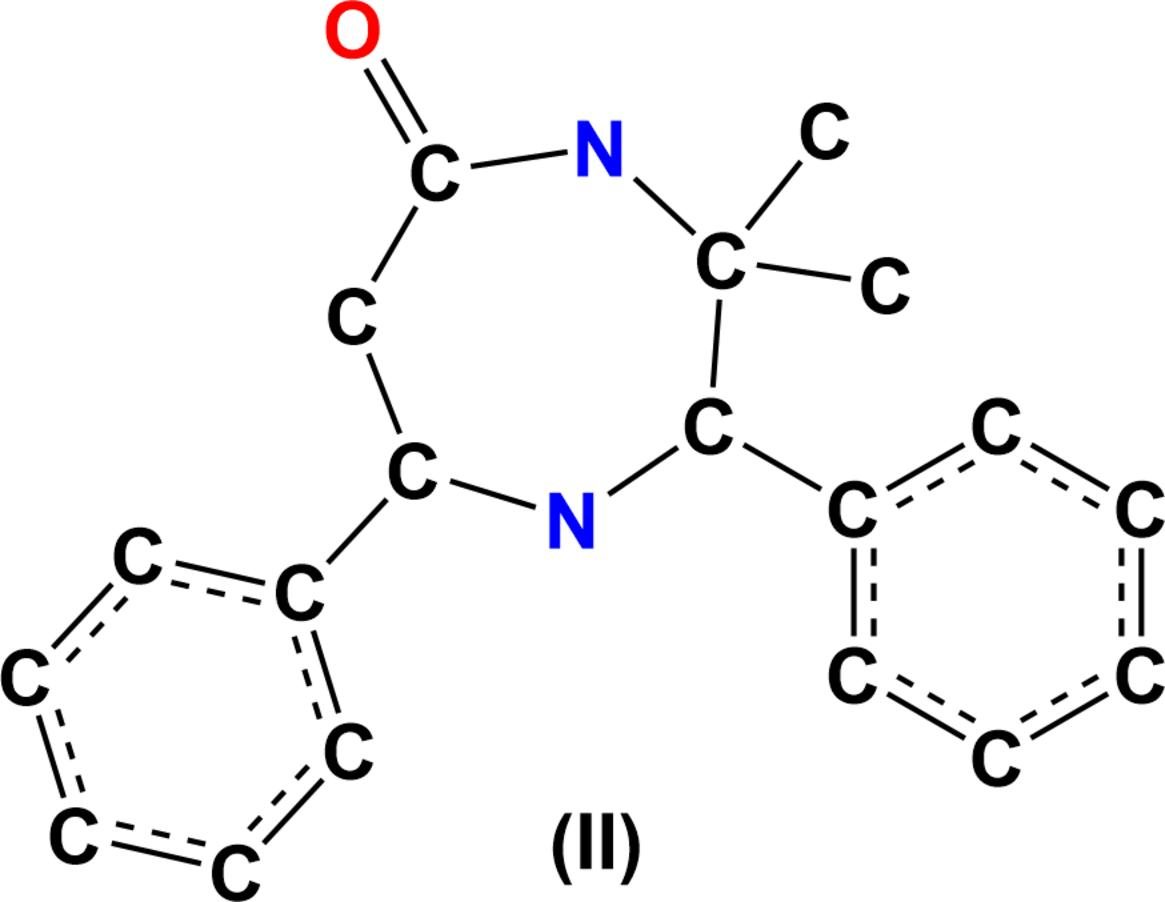
The mol­ecular moiety (II) used for the CSD database search.

A search using CCDC ConQuest of the Cambridge Structural Database (CSD, Version 5.44, updated to June 2023; Groom *et al.*, 2016 ▸) using the mol­ecular moiety (II) depicted in Fig. 10 ▸ for the basic skeleton of (I), omitting aromatic-H, methyl-H, methyl­ene-H, methine-H and Cl atoms gave five hits, *viz*. 3,3-dimethyl-1-nitroso-2,7-diphenyl-1,4-diazepan-5-one (CSD refcode KUZBUE; Ponnuswamy *et al.*, 2016 ▸), *c*-3,*t*-3-dimethyl-*r*-2,*c*-7-diphenyl-1,4-diazepan-5-one (PUGZAT; Ravichandran *et al.*, 2009 ▸), 3,3-dimethyl-2,7-bis­(4-methyl­phen­yl)-1,4-diazepan-5-one (QADRUL; Sethuvasan *et al.*, 2016 ▸), 2,7-bis­(2-chloro­phen­yl)-3,3-dimethyl-1,4-diazepan-5-one (QAD­SAS; Sethuvasan *et al.*, 2016 ▸) and 2,7-bis­(4-chloro­phen­yl)-3,3-dimethyl-1-nitroso-1,4-diazepan-5-one (WUPNED; Sethuvasan *et al.*, 2021 ▸).

The KUZBUE compound also has a chair conformation of the 1,4-diazepane group with diaxial phenyl groups. The structure of PUGZAT is closely related to that of the title compound having phenyl groups in place of the chloro­phenyl groups. In QADRUL, the planar 4-methyl­phenyl rings substituted at the C2 and C7 positions of the 1,4-diazepane ring, in a chair conformation, are in an equatorial orientation, as are the planar 2-chloro­phenyl rings substituted at these positions in QADSAS. On the other hand, in WUPNED, which is closely related to the title compound, both chloro­phenyl rings are in axial positions on the 1,4-diazepane chair ring. This makes a difference with the reported structure, where these substituents are in equatorial positions.

## 10. Synthesis and crystallization

The parent 2,6-bis­(4-chloro­phen­yl)-3,3-di­methyl­piperidin-4-one was prepared by double Mannich condensation of ethyl methyl ketone, 4-chloro­benzaldehyde and ammonium acetate in a 1:2:1 ratio following a previously reported procedure (Noller & Baliah, 1948 ▸). The title compound was obtained from the parent piperidin-4-one using a literature procedure (Thennarasu & Perumal, 2002 ▸). The compound was purified and single crystals suitable for X-ray analysis obtained by recrystallization from methanol using the slow evaporation technique (yield: 89%; m.p. 465 K).

## 11. Refinement

Crystal data, data collection and structure refinement details are summarized in Table 3 ▸. C-bound H atoms were placed in calculated positions (C—H = 0.93, 0.96, 0.97 and 0.98 Å for aromatic, methyl, methyl­ene and methine H atoms, respectively) and were included as riding contributions with isotropic displacement parameters *U*_iso_(H) = 1.2 and 1.5*U*_eq_(C). The H atoms attached to N1 and N4 were freely refined with N1—H1 = 0.854 (18) and N4—H4 = 0.86 (2) Å. The 4-chloro­phenyl ring at C7 is disordered over two positions with a refined occupancy ratio of 0.480 (16):0.520 (16) with an inter planar angle of 12.2 (4)°. Attempts to refine this model, including some geometric/ADP restraints (SAME, RIGU, SIMU and FLAT) were successful.

## Supplementary Material

Crystal structure: contains datablock(s) I. DOI: 10.1107/S2056989023010162/jw2001sup1.cif

Structure factors: contains datablock(s) I. DOI: 10.1107/S2056989023010162/jw2001Isup3.hkl

Supporting information file. DOI: 10.1107/S2056989023010162/jw2001Isup4.mol

Supporting information file. DOI: 10.1107/S2056989023010162/jw2001sup5.png

Supporting information file. DOI: 10.1107/S2056989023010162/jw2001sup6.png

Supporting information file. DOI: 10.1107/S2056989023010162/jw2001sup7.png

Supporting information file. DOI: 10.1107/S2056989023010162/jw2001Isup7.cml

CCDC reference: 2166707

Additional supporting information:  crystallographic information; 3D view; checkCIF report

## Figures and Tables

**Table 1:** Hydrogen-bond geometry (Å, °)

*D*—H⋯*A*	*D*—H	H⋯*A*	*D*⋯*A*	*D*—H⋯*A*
C32—H32*A*⋯O5^i^	0.96	2.57	3.253 (3)	129
C73*B*—H73*B*⋯O5^ii^	0.93	2.65	3.515 (13)	155
N4—H4⋯O5^i^	0.86 (2)	2.06 (2)	2.914 (2)	171.7 (17)

Symmetry codes: (i) 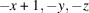 ; (ii) 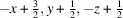 .

**Table 2:** Comparison of selected (X-ray and DFT) bond lengths, angles and torsion angles (Å, °)

	X-ray	B3LYP/6–31G(d,p)
N1—C2	1.470 (2)	1.470
C2—C3	1.564 (2)	1.575
C3—N4	1.483 (2)	1.479
N4—C5	1.344 (2)	1.372
C5—O5	1.232 (2)	1.227
C5—C6	1.514 (2)	1.522
C6—C7	1.535 (2)	1.544
C2—C21	1.525 (2)	1.524
C7—C71*B*	1.565 (10)	1.521
O5—C5—N4	120.56 (15)	120.0
O5—C5—C6	119.01 (15)	120.8
C7—N1—C2	115.87 (12)	117.0
N1—C2—C21	108.29 (12)	107.9
N1—C7—C71*B*	106.3 (4)	109.0
C21—C2—C3—N4	−166.89 (13)	−164.9
C5—C6—C7—C71*B*	163.1 (4)	164.0
N1—C2—C3—C31	−52.76 (19)	−52.2
N1—C2—C3—C32	−174.79 (15)	−173.8

**Table 3:** Experimental details

Crystal data	
Chemical formula	C_19_H_20_Cl_2_N_2_O
*M*_r_	363.27
Crystal system, space group	Monoclinic, *P*2_1_/*n*
Temperature (K)	303
*a*, *b*, *c* (Å)	12.364 (7), 11.148 (5), 13.898 (7)
β (°)	96.500 (19)
*V* (Å^3^)	1903.2 (17)
*Z*	4
Radiation type	Mo *K*α
μ (mm^−1^)	0.35
Crystal size (mm)	0.33 × 0.25 × 0.21

Data collection	
Diffractometer	Bruker D8 Quest XRD
Absorption correction	Multi-scan (*SADABS*; Krause *et al.*, 2015 ▸)
*T*_min_, *T*_max_	0.675, 0.746
No. of measured, independent and observed [*I* > 2σ(*I*)] reflections	26662, 5490, 3378
*R*_int_	0.037
(sin θ/λ)_max_ (Å^−1^)	0.703

Refinement	
*R*[*F* ^2^ > 2σ(*F* ^2^)], *wR*(*F* ^2^), *S*	0.049, 0.128, 1.03
No. of reflections	5490
No. of parameters	292
No. of restraints	308
H-atom treatment	H atoms treated by a mixture of independent and constrained refinement
Δρ_max_, Δρ_min_ (e Å^−3^)	0.27, −0.35

Computer programs: *APEX3* and *SAINT* (Bruker, 2017 ▸), *SHELXT* (Sheldrick, 2015*a* ▸), *SHELXL* (Sheldrick, 2015*b* ▸), *ORTEP-3 for Windows* (Farrugia, 2012 ▸), *PLATON* (Spek, 2020 ▸), *CrystalExplorer 21.5* (Spackman *et al.*, 2021 ▸) and *publCIF* (Westrip, 2010 ▸).

## supplementary crystallographic information

### Crystal data

**Table:**

C_19_H_20_Cl_2_N_2_O	*D*_x_ = 1.268 Mg m^−^^3^
*M**_r_* = 363.27	Melting point: 465 K
Monoclinic, *P*2_1_/*n*	Mo *K*α radiation, λ = 0.71073 Å
*a* = 12.364 (7) Å	Cell parameters from 7149 reflections
*b* = 11.148 (5) Å	θ = 2.3–27.9°
*c* = 13.898 (7) Å	µ = 0.35 mm^−^^1^
β = 96.500 (19)°	*T* = 303 K
*V* = 1903.2 (17) Å^3^	Block, colourless
*Z* = 4	0.33 × 0.25 × 0.21 mm
*F*(000) = 760	

### Data collection

**Table:**

Bruker D8 Quest XRD diffractometer	3378 reflections with *I* > 2σ(*I*)
Detector resolution: 7.3910 pixels mm^-1^	*R*_int_ = 0.037
ω and Phi Scans scans	θ_max_ = 30.0°, θ_min_ = 2.1°
Absorption correction: multi-scan (*SADABS*; Krause *et al.*, 2015)	*h* = −17→17
*T*_min_ = 0.675, *T*_max_ = 0.746	*k* = −15→15
26662 measured reflections	*l* = −19→19
5490 independent reflections	

### Refinement

**Table:**

Refinement on *F*^2^	Secondary atom site location: difference Fourier map
Least-squares matrix: full	Hydrogen site location: mixed
*R*[*F*^2^ > 2σ(*F*^2^)] = 0.049	H atoms treated by a mixture of independent and constrained refinement
*wR*(*F*^2^) = 0.128	*w* = 1/[σ^2^(*F*_o_^2^) + (0.0408*P*)^2^ + 0.5139*P*] where *P* = (*F*_o_^2^ + 2*F*_c_^2^)/3
*S* = 1.03	(Δ/σ)_max_ < 0.001
5490 reflections	Δρ_max_ = 0.27 e Å^−^^3^
292 parameters	Δρ_min_ = −0.35 e Å^−^^3^
308 restraints	Extinction correction: *SHELXL* (Sheldrick, 2015*b*), Fc^*^=kFc[1+0.001xFc^2^λ^3^/sin(2θ)]^-1/4^
Primary atom site location: structure-invariant direct methods	Extinction coefficient: 0.009 (2)

### Special details

**Table:**

Geometry. All esds (except the esd in the dihedral angle between two l.s. planes) are estimated using the full covariance matrix. The cell esds are taken into account individually in the estimation of esds in distances, angles and torsion angles; correlations between esds in cell parameters are only used when they are defined by crystal symmetry. An approximate (isotropic) treatment of cell esds is used for estimating esds involving l.s. planes.
Refinement. 1. Fixed Uiso At 1.2 times of: All C(H) groups, All C(H,H) groups At 1.5 times of: All C(H,H,H) groups 2. Restrained planarity Cl7A, C71A, C72A, C73A, C74A, C75A, C76A with sigma of 0.1 Cl7B, C71B, C72B, C73B, C74B, C75B, C76B with sigma of 0.1 3. Uiso/Uaniso restraints and constraints C71A ~ C72A ~ C73A ~ C74A ~ C75A ~ C76A ~ C71B ~ C72B ~ C73B ~ C74B ~ C75B ~ C76B: within 2A with sigma of 0.04 and sigma for terminal atoms of 0.08 within 2A 4. Rigid body (RIGU) restrains Cl7A, C71A, C72A, C73A, C74A, C75A, C76A, Cl7B, C71B, C72B, C73B, C74B, C75B, C76B with sigma for 1-2 distances of 0.004 and sigma for 1-3 distances of 0.004 5. Same fragment restrains {C21, C22, C23, C24, C25, C26} sigma for 1-2: 0.02, 1-3: 0.04 as in {C71B, C72B, C73B, C74B, C75B, C76B} {C21, C22, C23, C24, C25, C26} sigma for 1-2: 0.02, 1-3: 0.04 as in {C71A, C72A, C73A, C74A, C75A, C76A} 6. Others Sof(H7B)=Sof(Cl7B)=Sof(C71B)=Sof(C72B)=Sof(H72B)=Sof(C73B)=Sof(H73B)= Sof(C74B)=Sof(C75B)=Sof(H75B)=Sof(C76B)=Sof(H76B)=1-FVAR(1) Sof(H7A)=Sof(Cl7A)=Sof(C71A)=Sof(C72A)=Sof(H72A)=Sof(C73A)=Sof(H73A)= Sof(C74A)=Sof(C75A)=Sof(H75A)=Sof(C76A)=Sof(H76A)=FVAR(1) 7.a Ternary CH refined with riding coordinates: C2(H2), C7(H7A), C7(H7B) 7.b Secondary CH2 refined with riding coordinates: C6(H6A,H6B) 7.c Aromatic/amide H refined with riding coordinates: C22(H22), C23(H23), C25(H25), C26(H26), C72A(H72A), C73A(H73A), C75A(H75A), C76A(H76A), C72B(H72B), C73B(H73B), C75B(H75B), C76B(H76B) 7.d Idealised Me refined as rotating group: C31(H31A,H31B,H31C), C32(H32A,H32B,H32C)

### Fractional atomic coordinates and isotropic or equivalent isotropic displacement parameters (Å^2^)

**Table:**

	*x*	*y*	*z*	*U*_iso_*/*U*_eq_	Occ. (<1)
C2	0.42678 (13)	0.06408 (14)	0.29663 (12)	0.0505 (4)	
H2	0.497527	0.024372	0.310620	0.061*	
C3	0.37908 (15)	0.02934 (14)	0.19124 (12)	0.0563 (4)	
C5	0.50869 (14)	0.14605 (14)	0.09427 (12)	0.0531 (4)	
C6	0.50994 (15)	0.25904 (14)	0.15484 (12)	0.0572 (4)	
H6A	0.438954	0.296682	0.142841	0.069*	
H6B	0.562545	0.313999	0.132470	0.069*	
C7	0.53706 (13)	0.24325 (13)	0.26467 (12)	0.0514 (4)	
H7A	0.600394	0.190581	0.278399	0.062*	0.472 (16)
H7B	0.597421	0.186124	0.275903	0.062*	0.528 (16)
C21	0.35432 (13)	0.02263 (14)	0.37199 (11)	0.0500 (4)	
C22	0.38037 (16)	−0.07966 (16)	0.42716 (13)	0.0618 (4)	
H22	0.443332	−0.122056	0.418510	0.074*	
C23	0.31446 (17)	−0.11953 (18)	0.49463 (13)	0.0692 (5)	
H23	0.332555	−0.188505	0.530528	0.083*	
C24	0.22178 (16)	−0.05607 (18)	0.50812 (13)	0.0649 (5)	
C25	0.19441 (16)	0.04635 (17)	0.45575 (14)	0.0652 (5)	
H25	0.131943	0.089007	0.465544	0.078*	
C26	0.26095 (15)	0.08526 (15)	0.38826 (13)	0.0578 (4)	
H26	0.242727	0.154763	0.353105	0.069*	
C31	0.27622 (16)	0.0998 (2)	0.15445 (14)	0.0735 (6)	
H31A	0.291534	0.184181	0.158105	0.110*	
H31B	0.219379	0.081252	0.193735	0.110*	
H31C	0.253316	0.078017	0.088445	0.110*	
C32	0.3536 (2)	−0.10543 (17)	0.18778 (15)	0.0826 (7)	
H32A	0.330865	−0.128892	0.122133	0.124*	
H32B	0.296309	−0.122057	0.227101	0.124*	
H32C	0.417556	−0.149653	0.211950	0.124*	
N1	0.44413 (11)	0.19400 (12)	0.30811 (11)	0.0522 (3)	
N4	0.46228 (13)	0.04530 (13)	0.12336 (11)	0.0594 (4)	
O5	0.55447 (11)	0.14660 (11)	0.01998 (9)	0.0699 (4)	
Cl2	0.13826 (6)	−0.10582 (7)	0.59300 (5)	0.1053 (3)	
Cl7A	0.6660 (5)	0.7404 (5)	0.4033 (8)	0.1180 (17)	0.472 (16)
C71A	0.5662 (9)	0.3684 (10)	0.2958 (6)	0.0400 (14)	0.472 (16)
C72A	0.6732 (10)	0.3967 (13)	0.3197 (9)	0.066 (3)	0.472 (16)
H72A	0.726064	0.338329	0.314837	0.079*	0.472 (16)
C73A	0.7046 (12)	0.5102 (14)	0.3510 (11)	0.069 (3)	0.472 (16)
H73A	0.778174	0.528912	0.362774	0.083*	0.472 (16)
C74A	0.6290 (9)	0.5946 (11)	0.3646 (8)	0.063 (2)	0.472 (16)
C75A	0.5212 (7)	0.5719 (8)	0.3382 (9)	0.081 (2)	0.472 (16)
H75A	0.469022	0.631200	0.342252	0.097*	0.472 (16)
C76A	0.4918 (7)	0.4573 (7)	0.3050 (9)	0.070 (2)	0.472 (16)
H76A	0.418363	0.440537	0.288274	0.084*	0.472 (16)
Cl7B	0.6571 (4)	0.7280 (4)	0.4386 (5)	0.0942 (12)	0.528 (16)
C71B	0.5707 (8)	0.3609 (9)	0.3218 (6)	0.0416 (13)	0.528 (16)
C72B	0.6760 (9)	0.4015 (10)	0.3337 (8)	0.055 (2)	0.528 (16)
H72B	0.730714	0.352297	0.314940	0.066*	0.528 (16)
C73B	0.7039 (10)	0.5132 (11)	0.3726 (10)	0.0600 (19)	0.528 (16)
H73B	0.776377	0.536746	0.384069	0.072*	0.528 (16)
C74B	0.6232 (8)	0.5870 (9)	0.3936 (7)	0.0609 (18)	0.528 (16)
C75B	0.5194 (7)	0.5475 (7)	0.3872 (9)	0.088 (2)	0.528 (16)
H75B	0.465714	0.596690	0.407604	0.105*	0.528 (16)
C76B	0.4918 (6)	0.4342 (7)	0.3506 (8)	0.076 (2)	0.528 (16)
H76B	0.419875	0.408339	0.345728	0.092*	0.528 (16)
H1	0.4518 (14)	0.2083 (16)	0.3689 (14)	0.061 (5)*	
H4	0.4644 (15)	−0.0126 (17)	0.0823 (14)	0.067 (5)*	

### Atomic displacement parameters (Å^2^)

**Table:**

	*U*^11^	*U*^22^	*U*^33^	*U*^12^	*U*^13^	*U*^23^
C2	0.0524 (9)	0.0428 (8)	0.0567 (9)	−0.0010 (7)	0.0076 (7)	−0.0071 (7)
C3	0.0688 (11)	0.0474 (8)	0.0540 (9)	−0.0144 (8)	0.0131 (8)	−0.0106 (7)
C5	0.0575 (10)	0.0459 (8)	0.0556 (9)	−0.0056 (7)	0.0058 (8)	−0.0080 (7)
C6	0.0704 (11)	0.0412 (8)	0.0605 (10)	−0.0080 (7)	0.0101 (8)	−0.0074 (7)
C7	0.0502 (9)	0.0421 (8)	0.0616 (10)	−0.0035 (7)	0.0053 (7)	−0.0109 (7)
C21	0.0551 (10)	0.0450 (8)	0.0493 (8)	−0.0037 (7)	0.0040 (7)	−0.0086 (6)
C22	0.0653 (11)	0.0609 (10)	0.0590 (10)	0.0084 (8)	0.0058 (8)	0.0021 (8)
C23	0.0839 (14)	0.0688 (12)	0.0542 (10)	−0.0009 (10)	0.0049 (10)	0.0092 (9)
C24	0.0713 (12)	0.0744 (12)	0.0500 (9)	−0.0124 (10)	0.0112 (8)	−0.0072 (9)
C25	0.0648 (12)	0.0652 (11)	0.0678 (11)	0.0005 (9)	0.0164 (9)	−0.0125 (9)
C26	0.0650 (11)	0.0471 (9)	0.0620 (10)	0.0029 (8)	0.0103 (8)	−0.0062 (7)
C31	0.0649 (12)	0.0900 (14)	0.0633 (11)	−0.0162 (10)	−0.0020 (9)	−0.0043 (10)
C32	0.1246 (19)	0.0556 (11)	0.0731 (13)	−0.0334 (11)	0.0353 (12)	−0.0190 (9)
N1	0.0568 (8)	0.0451 (7)	0.0548 (8)	−0.0063 (6)	0.0077 (6)	−0.0136 (6)
N4	0.0808 (11)	0.0431 (7)	0.0574 (8)	−0.0107 (7)	0.0220 (7)	−0.0142 (6)
O5	0.0867 (9)	0.0602 (7)	0.0673 (8)	−0.0183 (6)	0.0290 (7)	−0.0156 (6)
Cl2	0.1109 (5)	0.1293 (6)	0.0826 (4)	−0.0138 (4)	0.0409 (4)	0.0141 (4)
Cl7A	0.1099 (17)	0.0723 (13)	0.175 (4)	−0.0380 (12)	0.029 (2)	−0.060 (2)
C71A	0.050 (2)	0.048 (2)	0.021 (3)	−0.0042 (17)	0.000 (3)	−0.004 (3)
C72A	0.060 (4)	0.052 (4)	0.089 (6)	−0.015 (3)	0.019 (4)	−0.023 (4)
C73A	0.059 (4)	0.064 (4)	0.083 (7)	−0.017 (3)	0.005 (4)	−0.017 (4)
C74A	0.070 (3)	0.054 (3)	0.065 (5)	−0.025 (2)	0.016 (3)	−0.027 (3)
C75A	0.062 (3)	0.057 (3)	0.126 (7)	−0.007 (2)	0.024 (4)	−0.037 (4)
C76A	0.046 (2)	0.056 (3)	0.108 (6)	−0.009 (2)	0.009 (4)	−0.029 (3)
Cl7B	0.0812 (15)	0.0640 (11)	0.133 (3)	−0.0084 (10)	−0.0070 (13)	−0.0489 (14)
C71B	0.055 (2)	0.044 (2)	0.024 (3)	−0.0014 (15)	−0.003 (2)	−0.005 (2)
C72B	0.048 (3)	0.050 (4)	0.067 (3)	0.001 (3)	0.002 (2)	0.000 (3)
C73B	0.055 (3)	0.057 (3)	0.066 (4)	−0.016 (2)	−0.004 (3)	−0.014 (3)
C74B	0.066 (3)	0.055 (3)	0.060 (4)	−0.002 (2)	−0.002 (3)	−0.022 (3)
C75B	0.063 (3)	0.067 (4)	0.132 (7)	−0.002 (2)	0.009 (4)	−0.046 (4)
C76B	0.054 (2)	0.060 (3)	0.115 (6)	−0.006 (2)	0.009 (4)	−0.036 (4)

### Geometric parameters (Å, º)

**Table:**

C2—N1	1.470 (2)	C31—H31B	0.9600
C2—C21	1.525 (2)	C31—H31C	0.9600
C2—C3	1.564 (2)	C32—H32A	0.9600
C2—H2	0.9800	C32—H32B	0.9600
C3—N4	1.483 (2)	C32—H32C	0.9600
C3—C31	1.532 (3)	N1—H1	0.854 (18)
C3—C32	1.535 (2)	N4—H4	0.86 (2)
C5—O5	1.232 (2)	Cl7A—C74A	1.756 (12)
C5—N4	1.344 (2)	C71A—C72A	1.364 (10)
C5—C6	1.514 (2)	C71A—C76A	1.369 (9)
C6—C7	1.535 (2)	C72A—C73A	1.379 (10)
C6—H6A	0.9700	C72A—H72A	0.9300
C6—H6B	0.9700	C73A—C74A	1.355 (10)
C7—N1	1.464 (2)	C73A—H73A	0.9300
C7—C71A	1.493 (11)	C74A—C75A	1.365 (9)
C7—C71B	1.565 (10)	C75A—C76A	1.392 (8)
C7—H7A	0.9800	C75A—H75A	0.9300
C7—H7B	0.9800	C76A—H76A	0.9300
C21—C26	1.389 (2)	Cl7B—C74B	1.726 (11)
C21—C22	1.391 (2)	C71B—C76B	1.367 (8)
C22—C23	1.383 (3)	C71B—C72B	1.371 (9)
C22—H22	0.9300	C72B—C73B	1.386 (9)
C23—C24	1.377 (3)	C72B—H72B	0.9300
C23—H23	0.9300	C73B—C74B	1.351 (9)
C24—C25	1.376 (3)	C73B—H73B	0.9300
C24—Cl2	1.744 (2)	C74B—C75B	1.350 (8)
C25—C26	1.385 (2)	C75B—C76B	1.390 (7)
C25—H25	0.9300	C75B—H75B	0.9300
C26—H26	0.9300	C76B—H76B	0.9300
C31—H31A	0.9600		
			
N1—C2—C21	108.29 (12)	H31A—C31—H31B	109.5
N1—C2—C3	112.36 (14)	C3—C31—H31C	109.5
C21—C2—C3	112.31 (13)	H31A—C31—H31C	109.5
N1—C2—H2	107.9	H31B—C31—H31C	109.5
C21—C2—H2	107.9	C3—C32—H32A	109.5
C3—C2—H2	107.9	C3—C32—H32B	109.5
N4—C3—C31	109.60 (15)	H32A—C32—H32B	109.5
N4—C3—C32	104.62 (14)	C3—C32—H32C	109.5
C31—C3—C32	109.34 (17)	H32A—C32—H32C	109.5
N4—C3—C2	110.54 (14)	H32B—C32—H32C	109.5
C31—C3—C2	113.34 (14)	C7—N1—C2	115.87 (12)
C32—C3—C2	109.02 (15)	C7—N1—H1	109.4 (12)
O5—C5—N4	120.56 (15)	C2—N1—H1	106.9 (12)
O5—C5—C6	119.01 (15)	C5—N4—C3	129.94 (14)
N4—C5—C6	120.38 (15)	C5—N4—H4	112.3 (13)
C5—C6—C7	116.40 (14)	C3—N4—H4	114.0 (12)
C5—C6—H6A	108.2	C72A—C71A—C76A	117.0 (9)
C7—C6—H6A	108.2	C72A—C71A—C7	118.9 (9)
C5—C6—H6B	108.2	C76A—C71A—C7	124.1 (8)
C7—C6—H6B	108.2	C71A—C72A—C73A	121.3 (11)
H6A—C6—H6B	107.3	C71A—C72A—H72A	119.4
N1—C7—C71A	114.0 (4)	C73A—C72A—H72A	119.4
N1—C7—C6	111.08 (14)	C74A—C73A—C72A	120.5 (11)
C71A—C7—C6	101.5 (4)	C74A—C73A—H73A	119.7
N1—C7—C71B	106.3 (4)	C72A—C73A—H73A	119.7
C6—C7—C71B	115.1 (3)	C73A—C74A—C75A	120.1 (9)
N1—C7—H7A	110.0	C73A—C74A—Cl7A	121.7 (9)
C71A—C7—H7A	110.0	C75A—C74A—Cl7A	117.8 (9)
C6—C7—H7A	110.0	C74A—C75A—C76A	118.0 (8)
N1—C7—H7B	108.0	C74A—C75A—H75A	121.0
C6—C7—H7B	108.0	C76A—C75A—H75A	121.0
C71B—C7—H7B	108.0	C71A—C76A—C75A	122.8 (8)
C26—C21—C22	117.83 (16)	C71A—C76A—H76A	118.6
C26—C21—C2	121.54 (15)	C75A—C76A—H76A	118.6
C22—C21—C2	120.63 (15)	C76B—C71B—C72B	117.9 (8)
C23—C22—C21	121.29 (17)	C76B—C71B—C7	119.5 (7)
C23—C22—H22	119.4	C72B—C71B—C7	122.1 (7)
C21—C22—H22	119.4	C71B—C72B—C73B	122.3 (9)
C24—C23—C22	119.35 (18)	C71B—C72B—H72B	118.9
C24—C23—H23	120.3	C73B—C72B—H72B	118.9
C22—C23—H23	120.3	C74B—C73B—C72B	118.4 (10)
C25—C24—C23	120.86 (17)	C74B—C73B—H73B	120.8
C25—C24—Cl2	119.52 (16)	C72B—C73B—H73B	120.8
C23—C24—Cl2	119.62 (16)	C75B—C74B—C73B	120.4 (9)
C24—C25—C26	119.25 (17)	C75B—C74B—Cl7B	120.8 (7)
C24—C25—H25	120.4	C73B—C74B—Cl7B	118.5 (8)
C26—C25—H25	120.4	C74B—C75B—C76B	120.9 (7)
C25—C26—C21	121.39 (17)	C74B—C75B—H75B	119.6
C25—C26—H26	119.3	C76B—C75B—H75B	119.6
C21—C26—H26	119.3	C71B—C76B—C75B	119.8 (7)
C3—C31—H31A	109.5	C71B—C76B—H76B	120.1
C3—C31—H31B	109.5	C75B—C76B—H76B	120.1
			
N1—C2—C3—N4	70.74 (17)	C31—C3—N4—C5	58.5 (2)
C21—C2—C3—N4	−166.89 (13)	C32—C3—N4—C5	175.64 (19)
N1—C2—C3—C31	−52.76 (19)	C2—C3—N4—C5	−67.1 (2)
C21—C2—C3—C31	69.62 (18)	N1—C7—C71A—C72A	135.9 (6)
N1—C2—C3—C32	−174.79 (15)	C6—C7—C71A—C72A	−104.6 (6)
C21—C2—C3—C32	−52.41 (19)	N1—C7—C71A—C76A	−42.0 (7)
O5—C5—C6—C7	−134.43 (18)	C6—C7—C71A—C76A	77.5 (6)
N4—C5—C6—C7	42.8 (2)	C76A—C71A—C72A—C73A	−0.5 (9)
C5—C6—C7—N1	−76.04 (18)	C7—C71A—C72A—C73A	−178.5 (8)
C5—C6—C7—C71A	162.5 (4)	C71A—C72A—C73A—C74A	4.2 (14)
C5—C6—C7—C71B	163.1 (4)	C72A—C73A—C74A—C75A	−6.7 (16)
N1—C2—C21—C26	45.9 (2)	C72A—C73A—C74A—Cl7A	−179.4 (7)
C3—C2—C21—C26	−78.75 (19)	C73A—C74A—C75A—C76A	5.3 (13)
N1—C2—C21—C22	−133.83 (16)	Cl7A—C74A—C75A—C76A	178.3 (5)
C3—C2—C21—C22	101.51 (18)	C72A—C71A—C76A—C75A	−0.8 (9)
C26—C21—C22—C23	1.4 (3)	C7—C71A—C76A—C75A	177.1 (6)
C2—C21—C22—C23	−178.88 (16)	C74A—C75A—C76A—C71A	−1.5 (10)
C21—C22—C23—C24	−0.6 (3)	N1—C7—C71B—C76B	−38.5 (6)
C22—C23—C24—C25	−0.3 (3)	C6—C7—C71B—C76B	85.0 (6)
C22—C23—C24—Cl2	179.97 (15)	N1—C7—C71B—C72B	150.0 (6)
C23—C24—C25—C26	0.4 (3)	C6—C7—C71B—C72B	−86.5 (6)
Cl2—C24—C25—C26	−179.84 (14)	C76B—C71B—C72B—C73B	−0.5 (8)
C24—C25—C26—C21	0.4 (3)	C7—C71B—C72B—C73B	171.1 (7)
C22—C21—C26—C25	−1.2 (3)	C71B—C72B—C73B—C74B	−4.4 (12)
C2—C21—C26—C25	179.01 (15)	C72B—C73B—C74B—C75B	7.6 (13)
C71A—C7—N1—C2	−167.4 (4)	C72B—C73B—C74B—Cl7B	−178.0 (7)
C6—C7—N1—C2	78.68 (18)	C73B—C74B—C75B—C76B	−6.0 (12)
C71B—C7—N1—C2	−155.4 (4)	Cl7B—C74B—C75B—C76B	179.7 (5)
C21—C2—N1—C7	161.36 (14)	C72B—C71B—C76B—C75B	2.3 (8)
C3—C2—N1—C7	−74.02 (19)	C7—C71B—C76B—C75B	−169.6 (6)
O5—C5—N4—C3	−161.09 (18)	C74B—C75B—C76B—C71B	0.9 (10)
C6—C5—N4—C3	21.7 (3)		

### Hydrogen-bond geometry (Å, º)

**Table:**

*D*—H···*A*	*D*—H	H···*A*	*D*···*A*	*D*—H···*A*
C32—H32*A*···O5^i^	0.96	2.57	3.253 (3)	129
C73*B*—H73*B*···O5^ii^	0.93	2.65	3.515 (13)	155
N4—H4···O5^i^	0.86 (2)	2.06 (2)	2.914 (2)	171.7 (17)

Symmetry codes: (i) −*x*+1, −*y*, −*z*; (ii) −*x*+3/2, *y*+1/2, −*z*+1/2.
