# Supplementary figures and images for: Synthesis, crystal structure and computational analysis of 2,7-bis­(4-chloro­phen­yl)-3,3-dimethyl-1,4-diazepan-5-one
Source: Acta Crystallogr E Crystallogr Commun. 2023 Nov 30;79(Pt 12):1212–7. doi: 10.1107/S2056989023010162 (PMC10833417; doi:10.1107/S2056989023010162)

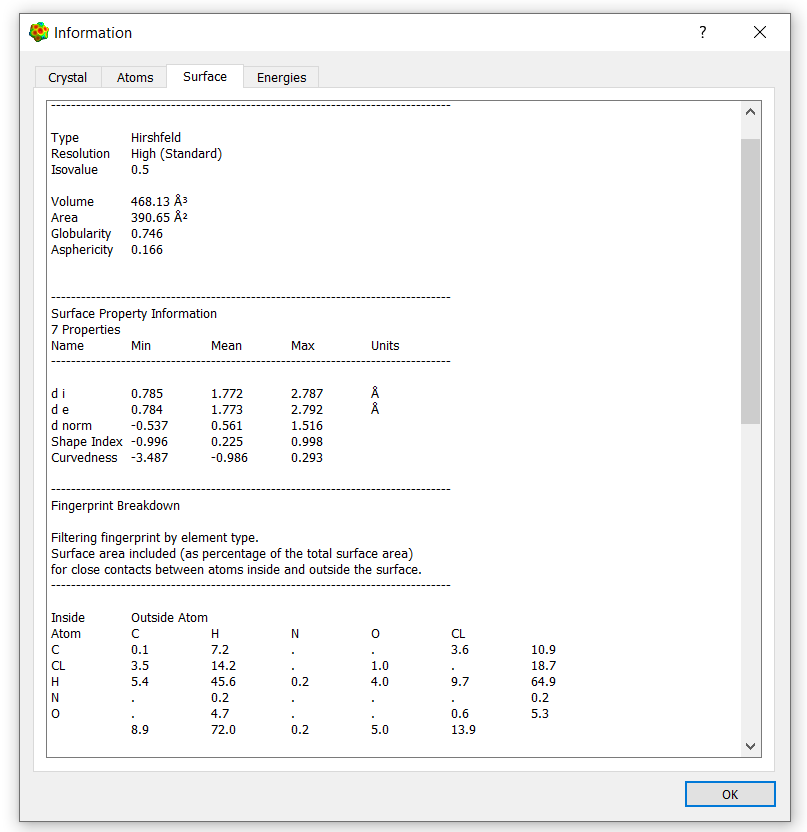

Supplement: Supplementary file 4 [file e-79-01212-sup5.png]

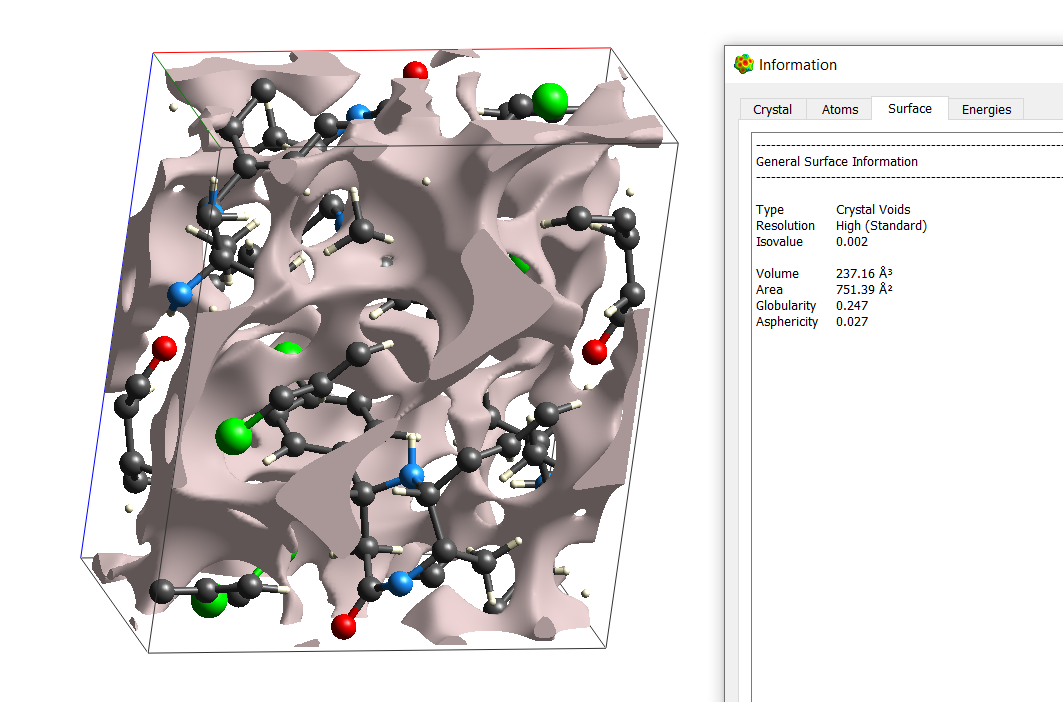

Supplement: Supplementary file 5 [file e-79-01212-sup6.png]

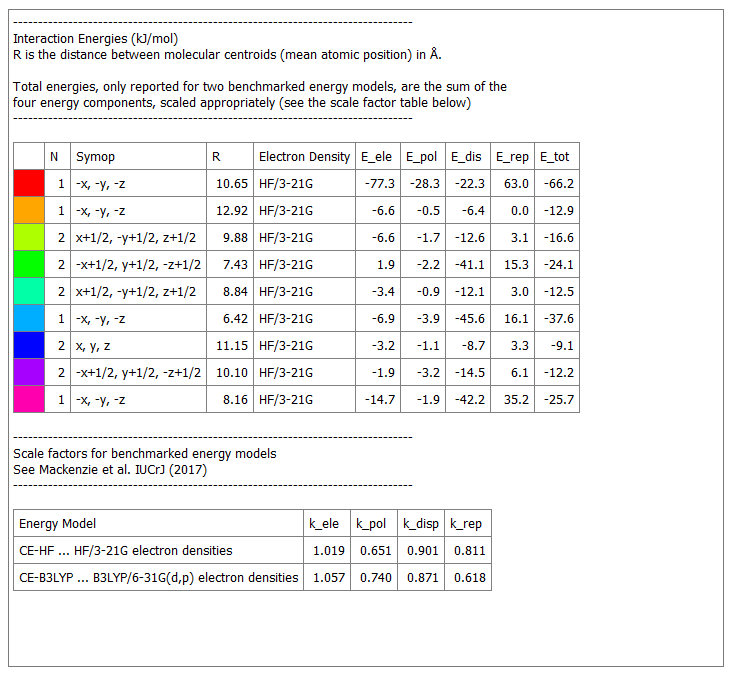

Supplement: Supplementary file 6 [file e-79-01212-sup7.png]
